# Asthma-Like Symptoms in Homeless Children in the Greater Paris Area in 2013: Prevalence, Associated Factors and Utilization of Healthcare Services in the ENFAMS Survey

**DOI:** 10.1371/journal.pone.0153872

**Published:** 2016-04-15

**Authors:** Delphine Lefeuvre, Marie-Christine Delmas, Christophe Marguet, Pierre Chauvin, Stéphanie Vandentorren

**Affiliations:** 1 INSERM, Sorbonne Universités, UPMC Univ Paris 06, Institut Pierre Louis d’Épidémiologie et de Santé Publique (IPLESP UMRS 1136), Department of social epidemiology, Paris, France; 2 French Institute for Public Health Surveillance, Saint-Maurice, France; 3 University Hospital of Rouen, Rouen, France; 4 Observatoire du Samusocial de Paris, Paris, France; Institute for Health & the Environment, UNITED STATES

## Abstract

**Introduction:**

Asthma remains poorly studied in homeless children. We sought to estimate the prevalence of asthma-like symptoms (ALS) and to identify the factors associated with ALS and healthcare service utilisation.

**Materials and Methods:**

A cross-sectional survey of a random sample of sheltered homeless families was conducted by interviewing 801 parents of children (0–12 years) in 17 languages. ALS were defined as wheezing or night cough without fever during the previous year. Poisson regression models with robust error variance were used to compute prevalence ratios (PR) for factors associated with ALS and healthcare service utilisation for ALS.

**Results:**

The prevalence of ALS among the children was 19.9%. Poor housing sanitation was significantly associated with ALS, as being born in the European Union. Most of the children with ALS had used healthcare services (85.4%). The main barriers to accessing such services were having lived in France for less than 49 months, having difficulties in French and living in poor housing conditions.

**Conclusion:**

ALS prevalence seemed lower than in the general child population, possibly because of the children's origins. Environmental factors associated with ALS point to the need to improve the indoor environment of family shelters. The relatively high rate of healthcare service utilisation should not overshadow existing barriers.

## Background

Asthma prevalence among children in the general population is approximately 10% in Western countries, such as the United States and France, but appears to be much lower in Africa and Northern and Eastern Europe [1, 2]. In the latest French national health surveys in schoolchildren, 9.8% of those aged 5 years and 14.4% of those aged 10 years had asthma. The prevalences of asthma-like symptoms (ALS) were, respectively, 10.7% and 10.1% for wheezing and 12.6% and 16.5% for night cough without fever [3, 4].

Asthma prevalence can reach as high as 40% among sheltered homeless children, according to several studies conducted in the United States [5–8]. Because homeless families are often housed in dilapidated buildings, they may be exposed to a poor indoor environment (moulds, dust mites, pets, mice, etc.), which is detrimental to their health, especially their respiratory health. Since the indoor environment is known to have a significant impact on childhood asthma [9–12], these factors deserve to be studied in this population. Also, since socioeconomic conditions and racial and ethnic disparities lead to inequalities in access to asthma treatment and care [13–17], homeless families may be at risk for under- or inappropriate healthcare service utilisation.

In 2013, given the dramatic increase in the number of homeless families in the Paris region over the previous ten years (exceeding that of lone homeless persons in 2010 [18]) and the complete lack of epidemiological data on homeless children, the ENFAMS survey (*Enfants et familles sans logement* [Homeless Children and Families]) specifically focused, for the first time in France, on sheltered homeless children and families in the Greater Paris Area.

The aims of our study were: 1) to estimate the prevalence of ALS among sheltered homeless children in this area; 2) to identify environmental and socioeconomic factors associated with ALS; and 3) to identify factors associated with healthcare service utilisation among children with ALS.

## Material and Methods

### The ENFAMS survey

The methodology used in the ENFAMS survey has been described elsewhere [19]. Briefly, the survey was conducted between January and May 2013 in a random sample of 801 homeless families with 566 children aged 0–5 years and 235 children aged 6–12 years in the Greater Paris Area (a region of 762 km^2^ with 6.7 million inhabitants). The survey employed a three-stage random sampling procedure. First, the sampling frame of the 796 existing accommodations for the homeless in the region were randomly selected using a stratification based on the type of facility, its distance from the centre of Paris and its distance from the nearest subway or railway station. Four types of facility have been considered: emergency housing centres which house homeless people or families for periods ranging from one night to a few months; social reinsertion centres which the objective is to help families to access or recover personal and social autonomy proposing individualised, globalised accompaniment for all families facing difficulties; centres for asylum-seekers which cater for people whilst their applications for asylum are being processed by the French refugee office; and social hotels which are part of the emergency housing centres initiative, but offer real private areas for each family (rooms with one or two beds instead of dormitories), a decent level of comfort, washing facilities and private kitchens or kitchens shared just by a few families. Second, 801 families were randomly selected from among those with at least one child under 13 years of age living with them. Third, one child under 13 was randomly selected. Family participation rate was 79%; non-participant adults were younger (mean age: 33 years vs. 38), more often men (15.3% of all those who refused vs. 4.6% of those who accepted) and had more than two children under care more frequently than participants (31.7% vs. 23.1%).

A face-to-face questionnaire was administered, in one of 17 languages, to the mother (or the father, if the mother was not there) by a trained interviewer and a psychologist. All questionnaires and information tools have been entirely translated from French into 8 languages (English, Arabic, Armenian, Bulgarian, Mongolian, Romanian, Russian, Tamil) by professional translators and validated by reverse translation. Then they were also translated orally in 8 other languages (Spanish, Italian, Portuguese, Serbian, Soninke, Wolof, Bambara and Lingala) by bilingual psychologists and survey interviewers. The questionnaire collected data on the parents' demographics, socioeconomic status, migration history, residential mobility and health, and the selected child’s demographics, health, diet, sleep patterns and physical activities.

The participants provided their written informed consent to participate for themselves and their child in accordance with French law. This study was approved by two ethics boards, the *Comité de protection des personnes* (CPP) in Île-de-France and the *Comité consultatitf sur le traitement de l'information en matière de recherche dans le domaine de la santé* (CCTIRS), and by the *Commission nationale de l'informatique et des libertés* (CNIL), which regulates individual data protection.

### Outcomes and explanatory variables

Based on the ISAAC questionnaire criteria [20], ALS were defined as wheezing and/or night cough without fever during the previous year.

Healthcare service utilisation (yes/no) was defined as any medical consultation, emergency department visit or hospitalisation for respiratory symptoms during the previous year. Children hospitalised for respiratory problems were considered severe cases.

Explanatory variables were the parents’ demographics (age, length of residence in France, and number of children), socioeconomic status (administrative status, education level, employment status, household income per consumption unit (CU), and difficulties in French) and health-related characteristics (self-reported physical health, smoking status, healthcare service utilisation [defined as at least one medical consultation during the previous 12 months], and health insurance status); housing characteristics (the type of homeless housing facility, poor sanitation including the presence of mice or cockroaches, cold, dampness, poor-quality bedding, a lack of space or crowding as reported by the parent, and the length of stay in the present housing facility); and the child’s demographics (age, sex and region of birth) and medical characteristics (atopic dermatitis, defined as an itchy rash having appeared and disappeared intermittently since birth, and the frequency of respiratory symptoms).

### Statistical analyses

**Fig 1:**
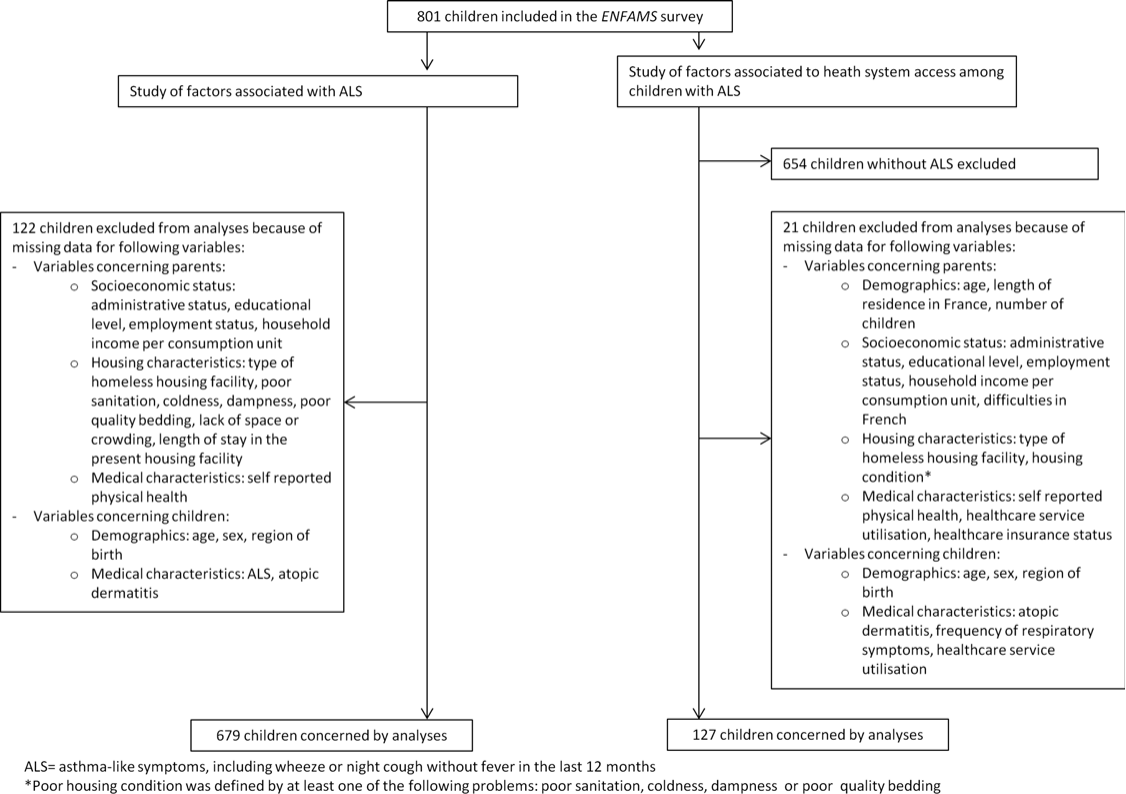
Flowchart.

Three populations were considered in our analysis. First, ALS prevalence was computed for all the children included in the ENFAMS survey. Second, the factors associated with ALS were analysed among the children with no missing data for any of these factors. Third, the factors associated with healthcare service utilisation were analysed among the children with ALS (again, those with no missing data) (Fig 1).

To account for the unequal probability of inclusion and to provide valid estimates, the complex sample design was redressed by weighting all our estimates and models by the inverse of the inclusion probability of each participant. Factors associated with previous-year ALS and healthcare service utilisation among children with ALS were explored using Poisson regression models, from which prevalence ratios (PRs) were obtained [21]. A stepwise variable selection procedure was performed to determine the final models (adding and removing thresholds of 0.10). Age was forced into both models, while sex was forced only into the analysis of factors associated with ALS. By way of an exploratory analysis of factors associated with ALS severity, we used the same procedure to study those associated with hospitalisation. All the analyses were performed using R software, version 3.1.2, with the survey package.

## Results

Eight hundred and one children were included in the ENFAMS survey, with an extrapolated estimate of 17,661 (95% CI = [16,353–18,969]) children under 13 housed with their parents in homeless facilities.

### Population’s characteristics

The children's median age was 4 years, 53.2% were girls, and 73% were born in the European Union (61.7% in France). For 95.3% of them, the interviewed parent was their mother (Table 1). Half of the children had a parent who was under 32 years of age, who had been living in France for less than 3.5 years and/or who reported a monthly household income under 227 €/CU. Two-thirds had parents who were legal residents (64.8%) but who had difficulties understanding, speaking, reading or writing French (64.9%). For the most part, the children were living with their parents in social hotels (76.5%), and this in poor conditions: 34.2% experienced cold, 36.5% experienced dampness and 45.5% experienced poor sanitation. They had been in their present housing facility for a median of 8 months.

**Table 1:** Population's characteristics.

	Children included in the study of ALS prevalence		Children included in the study of factors associated with ALS	Children with ALS included in the study of healthcare utilisation
	(N = 801)		(N = 679)	(N = 127)
	% or median [interquartile]	No. of missing data by variable	% or median [interquartile]	% or median [interquartile]
**Child characteristics**				
*Demographics*	* *	* *	* *	* *
Age		0		
0–1 year	29.0		30.7	36.9
2–4 years	30.3		28.3	33.1
5–12 years	40.7		41.0	30.1
Sex		0		
Boys	46.8		47.2	39.9
Girls	53.2		52.8	60.1
Region of birth		10		
European Union	73.0		73.1	80.7
Sub-Saharan Africa	9.1		9.2	4.6
CIS^1^ and other European countries	12.3		12.2	12.9
Other	5.5		5.5	1.7
*Medical characteristics*	* *	* *	* *	* *
Breastfeeding (0–5 years only)		22		
No	14.1		15.2	10.7
Yes	85.9		84.8	89.3
Age- and sex-adjusted BMI^2^		329		
Underweight	9.8		10.0	8.6
Normal	66.4		66.2	69.4
Overweight or obesity	23.8		23.8	22.1
Itchy rash		10		
No	65.4		65.2	51.9
Yes	34.6		34.8	48.1
Asthma-like symptoms (ALS)		11		
No	80.1		80.3	0.0
Yes	19.9		19.7	100.0
**Parental characteristics**				
*Demographics*	* *	* *	* *	* *
Sex		0		
Men	4.7		4.4	5.1
Women	95.3		95.6	94.9
Age (years)	32 [28–38]	0	32 [28–38]	32 [28–39]
Number of children living in the household		0		
1	25.2		26.1	19.2
2 or more	74.8		73.9	80.8
Length of residence in France (months)	42 [20–96]	40	42 [19–96]	49 [28–131]
*Socioeconomic status*	* *	* *	* *	* *
Education level		23		
None	11.2		10.4	7.3
Primary	12.6		12.8	8.6
Secondary	61.3		62.5	71.6
Tertiary	14.9		14.3	12.5
Difficulties in French		41		
No	35.1		36.5	41.8
Yes	64.9		63.5	58.2
Administrative status		2		
Regular	64.8		68	67.5
Irregular	35.2		32	32.5
Employment status		1		
Employed	19.9		20.0	16.1
Unemployed	29.7		30.6	30.4
Inactive^3^	50.4		49.4	53.5
Monthly household income (€ per CU^4^)	227 [52–518]	23	235 [52–526]	192 [63–539]
*Health-related characteristics*	* *	* *	* *	* *
Self-reported physical health status		4		
Poor or very poor	10.6		10.4	19.4
Very good, good or average	89.4		89.6	80.6
Smoking status		2		
Smoker	11.8		13.3	14.8
Non-smoker	88.2		86.7	85.2
Health insurance status		2		
None	19.2		19.1	19.4
Insurance for the poor or the undocumented	70.2		69.7	74.4
Usual Social security insurance	10.6		11.2	6.2
Healthcare service utilisation^5^		2		
No	20.5		19.9	16.9
Yes	79.5		80.1	83.1
Housing characteristics		0		
Type of homeless housing facility				
Centre for asylum-seekers	5.7		5.8	4.3
Social reinsertion centre	14.2		14.3	11.2
Emergency housing centre	3.7		3.4	3.7
Social hotel	76.5		76.5	80.8
Dampness		6		
No	63.5		62.4	45.1
Yes	36.5		37.6	54.9
Cold		3		
No	65.8		66.3	60.9
Yes	34.2		33.7	39.1
Sanitation^6^		8		
Good	54.5		54.4	38.4
Poor	45.5		45.6	61.6
Bedding quality		8		
Good	76.0		76.0	73.1
Poor	24.0		24.0	26.9
Lack of space or crowding		53		
No	77.2		77.2	79.9
Yes	22.8		22.8	20.1
Length of stay in the present housing facility (months)	8 [4–17]	1	8 [4–17]	8 [4–15]

^1^CIS: Commonwealth of Independent States

^2^BMI: body mass index

^3^Inactive: students, retirees, at home

^4^CU: consumption unit

^5^at least one medical consultation in the previous 12 months

^6^including the presence of mice and cockroaches.

### Weighted prevalence of wheezing, night cough without fever and either symptom (ALS)

**Fig 2:**
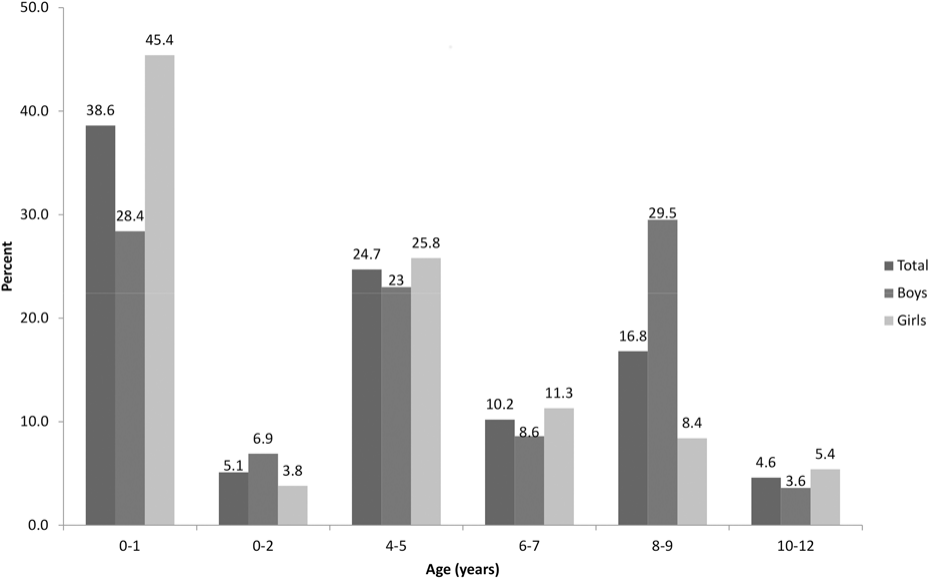
Age distribution of asthma-like symptoms, by gender, among the 801 children included in the ENFAMS survey (n = 147)

The data for wheezing, night cough without fever and either symptom concern, respectively, 792, 789 and 790 of the 810 children included in this survey. Among the respondents, the prevalence and 95% confidence intervals were 14.6% [10.9–18.2] for wheezing, 6.2% [4.1–8.2] for night cough without fever, and 19.9% [15.8–24.1] for either symptom (Table 2). Overall, these proportions seemed to be higher among girls than boys and varied with age, with the prevalence of wheezing being higher among the children aged 0–1 year and 6–7 years than in the other age groups. With regard to the children who had had either symptom, boys were more frequently affected than girls at age 8–9 compared to other age groups (Fig 2).

**Table 2:** Weighted prevalence of asthma-like symptoms (ALS).

	Wheezing (N = 792*)		Night cough without fever (N = 789*)		ALS (N = 790*)	
	n	% [95% CI]	n	% [95% CI]	n	% [95% CI]
Total	104	14.6 [10.9–18.2]	56	6.2 [4.1–8.2]	147	19.9 [15.8–24.1]
*By age*	* *	* *	* *	* *	* *	* *
0–1 year	38	20.4 [12.7–30.2]	19	6.8 [3.2–12.4]	54	26.0 [17.8–35.7]
2–3 years	28	16.0 [10.0–23.9]	14	7.1 [3.0–13.8]	39	22.0 [14.6–31.1]
4–5 years	14	8.1 [3.5–15.5]	7	5.2 [1.6–12.1]	19	13.1 [6.8–22.2]
6–7 years	11	17.1 [7.1–32.2]	8	6.9 [1.8–17.4]	16	23.1 [11.8–38.2]
8–9 years	5	4.9 [0.4–18.6]	4	5.7 [0.8–17.8]	8	10.3 [2.7–25.1]
10–12 years	8	9.0 [3.3–18.5]	4	2.7 [0.6–7.5]	11	11.1 [4.9–20.6]
*By sex*	* *	* *	* *	* *	* *	* *
Boys	47	14.1 [9.6–19.5]	20	4.0 [2.0–7.0]	62	17.0 [12.2–22.7]
Girls	57	15.1 [10.1–21.2]	36	8.1 [5.2–11.9]	85	22.6 [16.5–29.6]

ALS: asthma-like symptoms.

* Respective number of missing data: 9, 12 and 11.

### Factors associated with ALS

Of the 679 children included in the analysis (Fig 1), 19.6% (n = 126) had had ALS during the previous year. After adjustment for the children's age, sex and region of birth (children born outside the European Union being less frequently affected with ALS), poor sanitation was associated with ALS (PR = 1.54 [1.10–2.16]), while dampness was at the limit of the significance level (PR = 1.46 [0.98–2.16]). The children whose parents had poor or very poor self-reported physical health were also more frequently affected with ALS (PR = 1.68 [1.09–2.58]), such as those who ever had atopic dermatitis (PR = 1.96 [1.40–2.74]) (Table 3).

**Table 3:** Factors associated with asthma-like symptoms—univariate and multivariate analyses.

	% with ALS	Univariate analysis*	Multivariate analysis*	
	(N = 679)	PR [95% CI]	PR [95% CI]	p
*Child demographics*	* *	* *	* *	* *
Age				
0–1 year	27.0	Ref.	Ref.	0.05
2–4 years	22.4	0.83 [0.53–1.30]	0.97 [0.63–1.5]	
5–12 years	12.3	0.46 [0.26–0.80]	0.57 [0.35–0.94]	
Sex				
Boys	17.3	Ref.	Ref.	0.45
Girls	21.8	1.27 [0.85–1.88]	1.15 [0.80–1.64]	
Region of birth				
European Union	23.4	Ref.	Ref.	0.02
Sub-Saharan Africa	9.0	0.38 [0.22–0.67]	0.45 [0.24–0.84]	
CIS^1^ and other European countries	12.2	0.52 [0.24–1.14]	0.81 [0.39–1.68]	
Other	4.3	0.19 [0.06–0.57]	0.27 [0.09–0.83]	
*Housing characteristics*	* *	* *	* *	* *
Cold				
No	17.1	Ref.		
Yes	24.8	1.45 [0.94–2.24]		
Dampness				
No	14.6	Ref.	Ref.	0.06
Yes	28.2	1.94 [1.25–2.99]	1.46 [0.98–2.16]	
Sanitation (including the presence of mice and cockroaches)				
Good	14.0	Ref.	Ref.	0.01
Poor	26.5	1.89 [1.34–2.69]	1.54 [1.10–2.16]	
*Parental health-related characteristics *	* *	* *	* *	* *
Self-reported physical health				
Very good, good or average	17.7	Ref.	Ref.	0.02
Poor or very poor	36.4	2.05 [1.35–3.10]	1.68 [1.09–2.58]	
*Child medical characteristics*	* *	* *	* *	* *
Atopic dermatitis				
No	14.7	Ref.	Ref.	<0.01
Yes	29.1	1.98 [1.36–2.89]	1.96 [1.40–2.74]	

ALS: asthma-like symptoms; PR: prevalence ratio: 95% CI: 95% confidence interval.

*Poisson regression models with robust error variance. Only variables significant at p< 0.10 in univariate analysis are presented.

^1^CIS: Commonwealth of Independent States.

### Healthcare service utilisation for respiratory symptoms

One hundred and twenty-seven children with ALS were included in the analyses of the healthcare service utilisation for respiratory problems (Fig 1). Of them, 85.4% (n = 110) had used healthcare services during the previous year: 57.8% (n = 83) had been seen by a medical practitioner, 32.9% (n = 34) had visited an emergency department, and 32.1% (n = 35) had been hospitalised. Parental socioeconomic status and the child's region of birth were both significantly associated with healthcare service utilisation in bivariate analysis (Table 4). In the multivariate analysis adjusted for the children's age, those whose parents had been in France for less than 49 months or had difficulties in French and those who were living in poor housing conditions had used healthcare services less than the other children (PR: 0.84 [0.74–0.96], 0.87 [0.76–0.99] and 0.80 [0.67–0.96], respectively). The children whose parents had poor or very poor self-reported physical health or who had consulted a physician during the previous year used healthcare services more often than the other children with ALS. Also, the children with the least frequent symptoms (less than once a month) and those with the most frequent symptoms (more than once a week) had both used healthcare services more than the other children (PR: 1.51 [1.13–2.01] and 1.74 [1.30–2.34], respectively). The children who had had ALS more than once a week were also significantly at higher risk for hospitalisation than the reference group (PR = 4.71 [1.51–14.65]) (Table 5).

**Table 4:** Factors associated with healthcare service utilisation—univariate and multivariate analyses.

	% who used healthcare services	Univariate analysis*	Multivariate analysis*	
	(N = 127)	PR [95% CI]	PR [95% CI]	p
*Child demographics*	* *	* *	* *	* *
Age				
0–1 year	95.6	Ref.	Ref.	0.31
2–4 years	84.1	0.88 [0.74–1.05]	0.99 [0.87–1.14]	
5–12 years	74.3	0.78 [0.63–0.97]	0.89 [0.77–1.03]	
Region of birth				
European Union	90.9	Ref.		
Sub-Saharan Africa	47.3	0.52 [0.31–0.87]		
CIS^1^ and other European countries	65.0	0.72 [0.48–1.07]		
Other	81.2	0.89 [0.64–1.25]		
*Parental demographics*	* *	* *	* *	* *
Length of residence in France				
≥ 49 months	95.3	Ref.	Ref.	<0.01
< 49 months	75.1	0.79 [0.67–0.93]	0.84 [0.74–0.96]	
*Parental socioeconomic status*	* *	* *	* *	* *
Education level				
None	61.7	Ref.		
Primary	85.6	1.39 [0.78–2.46]		
Secondary	85.5	1.39 [0.80–2.41]		
Tertiary	98.1	1.59 [0.92–2.74]		
Difficulties in French				
No	95.2	Ref.	Ref.	0.04
Yes	78.3	0.82 [0.71–0.96]	0.87 [0.76–0.99]	
Administrative status				
Regular	94.4	Ref.		
Irregular	66.6	0.71 [0.55–0.9]		
Employment status				
Employed	100.0	Ref.		
Unemployed	82.6	0.83 [0.68–1]		
Inactive^2^	82.5	0.83 [0.73–0.93]		
Monthly household income (€ per CU^3^)				
< 192	79.4	Ref.		
≥ 192	91.0	1.15 [0.99–1.33]		
*Housing characteristics*	* *	* *	* *	* *
Housing conditions^4^				
Good	97.1	Ref.	Ref.	0.01
Poor	82.8	0.85 [0.76–0.95]	0.8 [0.67–0.96]	
*Parental health-related characteristics*				
Self-reported physical health				
Very good, good or average	81.9	Ref.	Ref.	<0.01
Poor or very poor	100.0	1.22 [1.11–1.35]	1.24 [1.08–1.43]	
Healthcare service utilisation (at least one medical consultation in the previous 12 months)				
No	45.2	Ref.	Ref.	<0.01
Yes	93.5	2.07 [1.22–3.53]	1.78 [1.17–2.71]	
*Child medical characteristics*				
Frequency of respiratory symptoms				
Less than once a month	90.5	1.73 [1.17–2.56]	1.51 [1.13–2.01]	<0.01
More than once a month but less than once a week	52.3	Ref.	Ref.	
More than once a week	99.1	1.89 [1.29–2.79]	1.74 [1.30–2.34]	

PR: prevalence ratio; 95% CI = 95% confidence interval.

*Poisson regression models with robust error variance. Only variables significant at p< 0.10 in univariate analysis are presented.

^1^CIS: Commonwealth of Independent States

^2^Inactive: students, retirees, at home

^3^CU: Consumption Unit

^4^Poor housing condition was defined by at least one of the following problem: poor sanitation, coldness, dampness or poor-quality bedding.

**Table 5:** Hospitalisations—bivariate and multivariate analyses (exploratory).

	% hospitalised	Univariate analysis*	Multivariate analysis*	
	(N = 127)	PR [95% CI]	PR [95% CI]	p
*Child demographics*		* *	* *	* *
Age				
0–1 year	37.5	Ref.	Ref.	0.13
2–4 years	16.3	0.43 [0.18–1.05]	0.73 [0.32–1.63]	
5–12 years	42.8	1.14 [0.6–2.17]	1.44 [0.8–2.6]	
*Parental socioeconomic status*		* *	* *	* *
Education level				
None	30.4	Ref.		
Primary	14.4	0.47 [0.09–2.39]		
Secondary	38.6	1.27 [0.46–3.48]		
Tertiary	8.1	0.27 [0.06–1.19]		
Administrative status				
Regular	38.6	Ref.		
Irregular	18.7	0.48 [0.23–1.01]		
Employment status				
Employed	60.5	Ref.	Ref.	0.01
Unemployed	37.8	0.62 [0.26–1.51]	0.67 [0.39–1.17]	
Inactive^1^	20.3	0.34 [0.15–0.77]	0.49 [0.29–0.82]	
*Parental health-related characteristics *				
Self-reported physical health				
Very good, good or average	62.6	Ref.	Ref.	<0.01
Poor or very poor	24.8	2.53 [1.46–4.37]	2.88 [1.57–5.29]	
*Child medical characteristics*		* *	* *	* *
Frequency of respiratory symptoms				
Less than once a month	31.3	3.00 [0.99–9.11]	2.19 [0.76–6.3]	0.01
More than once a month but less than once a week	10.4	Ref.	Ref.	
More than once a week	50.0	4.80 [1.54–14.93]	4.71 [1.51–14.65]	

PR: prevalence ratio; 95% CI: 95% confidence interval.

*Poisson regression models with robust error variance. Only variables significant at p<0.10 in univariate analysis are presented.

^1^Inactive: students, retirees, at home.

## Discussion

The ENFAMS survey, which was conducted in 17 languages, was the first one in France among homeless families, a hard-to-reach population. Its design made it possible to estimate ALS prevalence among homeless children housed in the Greater Paris Area and to identify associated factors, even if its cross-sectional design precludes any assertion of causality. The survey did not explicitly ask about asthma diagnosis because there is no consensual definition of asthma in preschool-aged children [22, 23].

We estimated that 19.9% of homeless children under 12 years of age in the Greater Paris Area had had ALS during the previous year. Those born in the European Union and those living in housing conditions with poor sanitation were more affected with ALS. Dampness was at the borderline of statistical significance. Even if most of the children with ALS had used healthcare, some barriers exist, such as a shorter parental length of residence in France, parental difficulties in French and poor housing conditions.

To the best of our knowledge, our study is the first one in France, as well as in Europe, to focus on the respiratory health of homeless children and their use of healthcare services.

**Fig 3:**
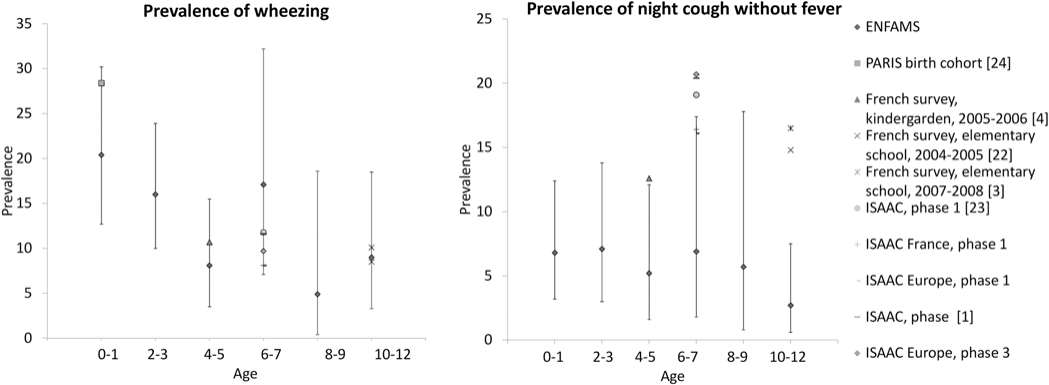
Prevalence of wheezing and night cough without fever—comparison between different studies.

Surprisingly, perhaps, the prevalence of ALS found in the ENFAMS survey tended to be lower than that observed in the general child population in previous French studies (Fig 3) [1, 3, 4, 24–26]. Indeed, only wheezing was more common in the ENFAMS survey, and then only among 6- and 7-year-olds. The fact that the survey was conducted in the winter may have resulted in a lower frequency of reported night cough without fever. Indeed, even though the parents were asked whether or not their child had usually had fever with his/her respiratory symptoms during the previous 12 months, we cannot rule out the possibility that they tended to answer in the affirmative because of a recent winter viral syndrome, failing to mention the previous episodes of respiratory symptoms (recall bias). Furthermore, it is known that the prevalence of asthma and ALS in immigrants from low- and middle income countries is lower than that in the host population of high-income countries [27–29]. In our study, 27% of the children were born outside the European Union in countries were asthma prevalence is, overall, lower than in France [1]. In the United States, where asthma prevalence in homeless children can reach 40%, the situation is completely different because most of them are African-American [5, 6], a subgroup of the population known for its high asthma prevalence compared to that among whites [2]).

Environmental triggers, such as second-hand smoke, and indoor allergens, such as mice, cockroaches and moulds, may increase asthma symptoms [9–12]. In the ENFAMS survey, dampness and poor sanitation were found to be associated with ALS. Of course, the data on ALS and indoor environments may have been biased in the ENFAMS survey, since they were reported by the parents. However, it was previously found that parental reports regarding exposure to dampness were more reliable than onsite inspections and that self-reported allergic symptoms were consistent with medical examinations [30]. Unfortunately, these asthma triggers are frequently encountered in homeless shelter environments, but the ability of families in such facilities to control their living environment is limited [31]. On the subject of tobacco smoke, the smoking status only of the interviewed parent (the mother in most cases) was asked about, so we did not have an accurate measurement of the children’s exposure.

Poor parental self-reported health may have had an effect on the answers regarding the children's health, but ALS does not seem to have been overestimated in the ENFAMS survey. Indeed, the children with ALS had had atopic dermatitis more frequently, which is consistent with the literature on asthma and wheezing illness. It will be noted that atopic dermatitis in early childhood seems to increase the risk of asthma later in childhood [32]. In many school-aged asthmatic children, wheezing occurs before or with the onset of atopic dermatitis [33]. As well, atopic dermatitis may have been overreported in the survey (it was defined as “itchy rashes appearing and disappearing intermittently since birth”), but it seems unlikely that it was reported differently between the children with and without ALS.

No socioeconomic factors were associated with ALS in the ENFAMS survey, in which the families had a very low household income and were often unemployed but had a relatively good education level. Actually, associations between these factors and asthma or wheezing illness are found inconsistently in the literature [4, 34–37]. Furthermore, the prevalence of wheezing and asthma is consistently reported to be higher in boys than in girls [4, 38, 39]. In our study, this difference was observed in 8- and 9-year-olds only. This could reflect the predominant role of housing and living conditions in this underprivileged population, as no difference was found in a U.S. study either, in homeless children aged 4 to 7 years [7].

Eventually, we cannot highlight in the present study the role of the stress as a contributor to ALS or to exacerbation of symptoms among children already at risk for ALS [40] when homeless children living in housing conditions are known to be exposed to many of stressors, such as community and family violence [41]. Also, another limit of our study is the absence of distinction between atopic and non atopic asthma phenotypes. Risk factors and measures of severity vary between children with different asthma phenotypes [42] but the data not allowed us to distinguish these phenotypes (the dermatitis atopic was probably not well declared and there was no other sign of atopy requested).

Missing data were taking into account using a listwise deletion method. In the analysis of factors associated with ALS, 679 children were included and 122 excluded (15.2%). There was no notable selection bias (in particular no difference in ALS prevalence), except that included children had parents who were more often in regular situation (68.0% versus 47.8%, p<0.01) and were more frequently smoker (13.3 versus 3.6%, p<0.01). In the analysis of the healthcare service utilisation for respiratory problems, 127 children with ALS were included and 20 excluded (13.6%). Included children had more frequent respiratory symptoms (25.9% had symptoms more than once a week, versus 3.8% of the excluded children, p = 0.03), used less healthcare service (85.4% versus 96.8%, p = 0.05), and had parents with a higher monthly household income per consumption unit (332.3 +/- 43.3€ versus 185.1 +/- 43.0€, p = 0.02) but with a more frequent lack of any health insurance (p<0.01).

The overall high proportion of children with ALS who had used healthcare services at least once during the previous year may seem encouraging, but only 57.8% of them had actually been seen by a medical practitioner, which could indicate a lack of regular follow-up. Inequalities in asthma care have been reported among minority and poor children, who are at greater risk for undertreatment, suboptimal disease control, emergency healthcare utilisation and hospitalisation [5–7, 13–16]. In France, according to a national survey among 5-year-olds, 4.6% of those with wheezing or being treated for wheezing or asthma (n = 2237) had been hospitalised during the year preceding the survey [4]. In the ENFAMS survey, the hospitalisation rate for ALS was 32.1%.

Our data suggest a socioeconomic gradient in healthcare utilisation by children with ALS in this population. In univariate analysis, a low level of education, being unemployed or inactive, an irregular administrative status, and a low or very low income are all parental factors associated with lower healthcare service utilisation by their children. A lack of statistical power may explain why this was not observed in multivariate analysis. However, in the multivariate model, a shorter length of residence in France, difficulties in French and parental non-use of healthcare services remained as barriers to accessing healthcare. This may reflect a lower level of acculturation and of knowledge concerning healthcare norms and the organisation of healthcare services in France [43].

## Conclusion

ALS prevalence in homeless children seems to be lower than that observed in the general child population. This may be due to the origin of some of these children. Environmental factors associated with ALS, such as dampness and poor housing sanitation, point to the need to improve the indoor environment of family shelters in order to limit asthma triggers. Despite a relatively high rate of healthcare service utilisation for ALS, there are still barriers to accessing them for the most underprivileged and/or the least acculturated migrants that need be addressed to improve care for this vulnerable child population.

## References

[1] Pearce N, Aït-Khaled N, Beasley R, Mallol J, Keil U, Mitchell E, et al Worldwide trends in the prevalence of asthma symptoms: phase III of the International Study of Asthma and Allergies in Childhood (ISAAC). Thorax 2007; 62: 758–766. 17504817 10.1136/thx.2006.070169 PMC2117323

[2] Akinbami L, Moorman J, Liu X. Asthma Prevalence, Health Care Use, and Mortality: United States, 2005–2009. National health statistics reports; no32. Hyattsville, MD, National Center for Health Statistics; 2011; 15p. 21355352

[3] Delmas MC, Guignon N, Leynaert B, Com-Ruelle L, Annesi-Maesano I, Chardon O, et al Évolution de la prévalence de l’asthme chez l’enfant en France: enquêtes nationales de santé en milieu scolaire 2003–2008. Bull Epidémiol Hebd 2014; 20: 360–365.

[4] Delmas M-C, Guignon N, Leynaert B, Annesi-Maesano I, Com-Ruelle L, Gonzalez L, et al Prévalence et contrôle de l’asthme chez le jeune enfant en France. Rev Mal Respir 2012; 29: 688–696. 10.1016/j.rmr.2011.11.016 22682595

[5] McLean DE, Bowen S, Drezner K, Rowe A, Sherman P, Schroeder S, et al Asthma among homeless children: undercounting and undertreating the underserved. Arch Pediatr Adolesc Med 2004; 158: 244–249. 14993083 10.1001/archpedi.158.3.244

[6] Grant R, Bowen S, McLean DE, Berman D, Redlener K, Redlener I. Asthma among homeless children in New York City: an update. Am J Public Health 2007; 97: 448–450. 17267730 10.2105/AJPH.2005.070482 PMC1805027

[7] Cutuli JJ, Herbers JE, Rinaldi M, Masten AS, Oberg CN. Asthma and behavior in homeless 4- to 7-year-olds. Pediatrics 2010; 125: 145–151. 10.1542/peds.2009-0103 19969617

[8] Cutuli JJ, Herbers JE, Lafavor TL, Ahumada SM, Masten AS, Oberg CN. Asthma and adaptive functioning among homeless kindergarten-aged children in emergency housing. J Health Care Poor Underserved 2014; 25: 717–730. 10.1353/hpu.2014.0099 24858881 PMC4498570

[9] Simoni M, Lombardi E, Berti G, Rusconi F, La Grutta S, Piffer S, et al Mould/dampness exposure at home is associated with respiratory disorders in Italian children and adolescents: the SIDRIA-2 Study. Occup Environ Med 2005; 62: 616–622. 16109818 10.1136/oem.2004.018291 PMC1741087

[10] Venn AJ, Cooper M, Antoniak M, Laughlin C, Britton J, Lewis SA. Effects of volatile organic compounds, damp, and other environmental exposures in the home on wheezing illness in children. Thorax 2003; 58: 955–960. 14586048 10.1136/thorax.58.11.955 PMC1746513

[11] Sun Y, Sundell J. Life style and home environment are associated with racial disparities of asthma and allergy in Northeast Texas children. Sci Total Environ 2011; 409: 4229–4234. 10.1016/j.scitotenv.2011.07.011 21802705

[12] Ahluwalia SK, Matsui EC. The indoor environment and its effects on childhood asthma. Curr Opin Allergy Clin Immunol 2011; 11: 137–143. 10.1097/ACI.0b013e3283445921 21301330

[13] Crocker D, Brown C, Moolenaar R, Moorman J, Bailey C, Mannino D, et al Racial and ethnic disparities in asthma medication usage and health-care utilization: Data from the national asthma survey. Chest 2009; 136: 1063–1071. 10.1378/chest.09-0013 19567492

[14] Flores G, Snowden-Bridon C, Torres S, Perez R, Walter T, Brotanek J, et al Urban minority children with asthma: substantial morbidity, compromised quality and access to specialists, and the importance of poverty and specialty care. J Asthma 2009; 46: 392–398. 10.1080/02770900802712971 19484676

[15] Stingone JA, Claudio L. Disparities in the use of urgent health care services among asthmatic children. Ann Allergy Asthma Immunol 2006; 97: 244–250. 16937759 10.1016/S1081-1206(10)60021-X

[16] Cantarero-Arévalo L, Holstein BE, Andersen A, Kaae S, Nørredam M, Hansen EH. Inequalities in asthma treatment among children by country of birth and ancestry: a nationwide study in Denmark. J Epidemiol Community Health 2013; 67: 912–917. 10.1136/jech-2012-202135 23814271

[17] Fung V, Graetz I, Galbraith A, Hamity C, Huang J, Vollmer WM, et al Financial barriers to care among low-income children with asthma: health care reform implications. JAMA Pediatr 2014; 168: 649–656. 10.1001/jamapediatrics.2014.79 24840805 PMC7105170

[18] Guyavarch E, Garcin E. Publics hébergés par le 115 de Paris: une forte progression des familles. Informations Sociales 2014; 182: 142–149.

[19] Vandentorren S, Le Méner E, Oppenchaim N, Arnaud A, Jangal C, Caum C, et al Characteristics and health of homeless families: the ENFAMS survey in the Paris region, France 2013. Eur J Public Health 2015, 10.1093/eurpub/ckv187 26511600

[20] Asher MI, Keil U, Anderson HR, Beasley R, Crane J, Martinez F, et al International Study of Asthma and Allergies in Childhood (ISAAC): rationale and methods. Eur. Respir. J. 1995; 8: 483–491. 7789502 10.1183/09031936.95.08030483

[21] Zou G. A Modified Poisson Regression Approach to Prospective Studies with Binary Data. Am J Epidemiol 2004; 159: 702–706. 15033648 10.1093/aje/kwh090

[22] Strunk RC. Defining asthma in the preschool-aged child. Pediatrics 2002; 109: 357–361. 11826250

[23] Société Pédiatrique de Pneumologie et Allergologie. Asthme de l’enfant de moins de 36 mois: diagnostic, prise en charge et traitement en dehors des épisodes aigus—Recommandations professionnelles. Saint-Denis: Haute Autorité de Santé; 2009, 24p.

[24] Delmas M-C, Fuhrman C. L’asthme en France: synthèse des données épidémiologiques descriptives. Rev Mal Respir 2010; 27: 151–159. 10.1016/j.rmr.2009.09.001 20206063

[25] Worldwide variations in the prevalence of asthma symptoms: the International Study of Asthma and Allergies in Childhood (ISAAC). Eur Respir J 1998; 12: 315–335. 9727780 10.1183/09031936.98.12020315

[26] Herr M, Nikasinovic L, Foucault C, Le Marec AM, Giordanella JP, Just J, et al Prise en charge des sifflements chez le nourrisson dans la cohorte de nouveau-nés PARIS. Rev Mal Respir 2012; 29: 52–59. 10.1016/j.rmr.2011.05.016 22240220

[27] Bråbäck L, Vogt H, Hjern A. Migration and asthma medication in international adoptees and immigrant families in Sweden. Clin Exp Allergy 2011; 41: 1108–1115. 10.1111/j.1365-2222.2011.03744.x 21481023

[28] Cabieses B, Uphoff E, Pinart M, Antó JM, Wright J. A systematic review on the development of asthma and allergic diseases in relation to international immigration: the leading role of the environment confirmed. PLoS One 2014; 9: e105347 10.1371/journal.pone.0105347 25141011 PMC4139367

[29] Garcia-Marcos L, Robertson CF, Ross Anderson H, Ellwood P, Williams HC, Wong GW, et al Does migration affect asthma, rhinoconjunctivitis and eczema prevalence? Global findings from the international study of asthma and allergies in childhood. Int J Epidemiol 2014; 43: 1846–1854. 10.1093/ije/dyu145 25056339

[30] Sun Y, Sundell J, Zhang Y. Validity of building characteristics and dorm dampness obtained in a self-administrated questionnaire. Sci Total Environ 2007; 387: 276–282. 17692898 10.1016/j.scitotenv.2007.07.001

[31] Buu MC, Carter L, Bruce JS, Baca EA, Greenberg B, Chamberlain LJ. Asthma, tobacco smoke and the indoor environment: a qualitative study of sheltered homeless families. J Asthma 2014; 51: 142–148. 10.3109/02770903.2013.857682 24147583

[32] Van der Hulst AE, Klip H, Brand PLP. Risk of developing asthma in young children with atopic eczema: a systematic review. J Allergy Clin Immunol 2007; 120: 565–569. 17655920 10.1016/j.jaci.2007.05.042

[33] Illi S, von Mutius E, Lau S, Nickel R, Grüber C, Niggemann B, et al The natural course of atopic dermatitis from birth to age 7 years and the association with asthma. J Allergy Clin Immunol 2004; 113: 925–931. 15131576 10.1016/j.jaci.2004.01.778

[34] Victorino CC, Gauthier AH. The social determinants of child health: variations across health outcomes—a population-based cross-sectional analysis. BMC Pediatr 2009; 9: 53 10.1186/1471-2431-9-53 19686599 PMC2734529

[35] Currie A, Shields MA, Price SW. The child health/family income gradient: Evidence from England. J Health Econ 2007; 26: 213–232. 16962191 10.1016/j.jhealeco.2006.08.003

[36] Hammer-Helmich L, Linneberg A, Thomsen SF, Glümer C. Association between parental socioeconomic position and prevalence of asthma, atopic eczema and hay fever in children. Scand J Public Health 2014; 42: 120–127. 10.1177/1403494813505727 24089102

[37] Gehring U, Pattenden S, Slachtova H, Antova T, Braun-Fahrländer C, Fabianova E, et al Parental education and children’s respiratory and allergic symptoms in the Pollution and the Young (PATY) study. Eur Respir J 2006; 27: 95–107. 16387941 10.1183/09031936.06.00017205

[38] Almqvist C, Worm M, Leynaert B, for the working group of GA2LEN WP 2.5 “Gender.” Impact of gender on asthma in childhood and adolescence: a GA2LEN review. Allergy 2008; 63: 47–57. 17822448 10.1111/j.1398-9995.2007.01524.x

[39] Delmas M-C, Guignon N, Leynaert B, Com-Ruelle L, Annesi-Maesano I, Herbet J-B, et al Prévalence de l’asthme chez l’enfant en France. Arch Pediatr 2009; 16: 1261–1269. 10.1016/j.arcped.2009.06.009 19625171

[40] Chen E, Miller GE. Stress and Inflammation in Exacerbations of Asthma. Brain Behav Immun 2007; 21: 993–999. 17493786 10.1016/j.bbi.2007.03.009 PMC2077080

[41] Samuels J, Shinn M, Buckner JC. Homeless Children: Update on Research, Policy, Programs, and Opportunities. Office of the Assistant Secretary for Planning and Evaluation; 2010.

[42] Kelley CF, Mannino DM, Homa DM, Savage-Brown A, Holguin F. Asthma Phenotypes, Risk Factors, and Measures of Severity in a National Sample of US Children. Pediatrics 2005; 115: 726–731. 15741378 10.1542/peds.2004-0529

[43] Berchet C, Jusot F. État de santé et recours aux soins des immigrés en France: une revue de la littérature. Bull Epidemiol Hebd 2012; 17–21.

